# Effects of meloxicam administration at birth on blood gas, acid-base status, and predictive value of early cardiac autonomic variables for postnatal adaptation in Holstein-Friesian calves

**DOI:** 10.3389/fvets.2026.1924579

**Published:** 2026-08-31

**Authors:** Levente Kovács, Fruzsina Luca Kézér, Zsófia Nyerges-Bohák, Ottó Szenci

**Affiliations:** 1 Department of Animal Husbandry and Animal Welfare, Institute of Animal Sciences, Hungarian University of Agriculture and Life Sciences, Gödöllo, Hungary; 2 Bona Adventure Ltd., Gödöllo, Hungary; 3 Department of Obstetrics and Food Animal Medicine Clinic, University of Veterinary Medicine Budapest, Üllo, Hungary

**Keywords:** acid-base, blood gas, calving ease, heart rate variability, meloxicam, neonatal calf, parity, vitality

## Abstract

**Background:**

Non-steroidal anti-inflammatory drugs (NSAIDs) are increasingly administered at birth to neonatal dairy calves, yet the interaction between periparturient meloxicam treatment and calving difficulty on vitality recovery and acid-base normalization has not been prospectively examined. The predictive value of early cardiac autonomic variables for compromised postnatal adaptation also remains unclear.

**Methods:**

In a quasi-randomized, blinded, placebo-controlled trial on 151 Holstein-Friesian calves (NSAID *n* = 54, Placebo *n* = 97), a single subcutaneous dose of meloxicam (0.5 mg/kg) or visually identical placebo was administered immediately after delivery. Neonatal vitality was scored at 0, 1, 2, 24, and 48 h using a validated 12-point scale. Venous blood gas and acid-base parameters were analyzed at 0, 1, 2, and 24 h. Heart rate variability (HRV) parameters were calculated from 5-min recording windows at 5 and 30 min postpartum. Linear mixed models (LMM) evaluated vitality and blood gas dynamics. Logistic regression and ROC analysis assessed the predictive value of early HRV and calving difficulty for low vitality at 2 h, including a multivariable model testing the incremental value of HRV beyond calving difficulty.

**Results:**

A significant three-way interaction (NSAID × time × calving difficulty) was detected (χ^2^ = 85.2, *P* < 0.001; *R*^2^*m* = 0.318); NSAID-treated calves had higher vitality at 24 h (*P* = 0.026) and 48 h (*P* = 0.017), most pronounced in dystocic calves. Blood gas parameters did not differ at birth; base excess (BE) was modestly lower in NSAID calves at 24 h (4.45 vs. 5.30 mmol/L; *P* = 0.049), although this was not supported in the repeated-measures model. Ionized calcium was lower in NSAID calves at 0 h and 24 h (*P* < 0.01). In the Placebo group, dam parity was significantly associated with HRV at 30 min (RMSSD rho = 0.50, *P* < 0.001). RMSSD at 5 min was the only HRV variable significantly associated with low vitality at 2 h (*AUC* = 0.635; *P* = 0.004). Calving difficulty was the strongest predictor (*OR* = 3.54 per unit; *AUC* = 0.772; *P* < 0.001); adding RMSSD to a calving difficulty model improved model fit but did not improve discrimination (Δ*AUC* = +0.027, 95% *CI* −0.025 to 0.075; *P* = 0.300).

**Conclusion:**

Periparturient meloxicam may modulate neonatal vitality recovery in a calving difficulty-dependent manner without directly reversing perinatal acidosis. Early HRV does not provide clinically useful prediction of compromised postnatal adaptation, and adds no incremental discriminative value beyond calving difficulty; calving difficulty score is the primary determinant. These findings suggest that targeted periparturient NSAID administration may be most justified for calves born after obstetrical assistance, although the quasi-randomized design warrants confirmation in a fully randomized trial.

## 1 Introduction

The neonatal period constitutes a critical transition window during which the dairy calf must rapidly adapt from intrauterine to extrauterine conditions. This transition is characterized by profound shifts in respiratory function, autonomic regulation, acid-base balance, and thermoregulation, all of which must be completed within the first hours of life to ensure survival (1, 2). The degree of physiological compromise during this period is strongly influenced by the obstetrical circumstances of delivery; dystocic calves experience extended periods of relative fetal hypoxia and acidosis that may impair postnatal adaptation, as reflected by lower neonatal vitality scores, more severe respiratory and metabolic acidosis, and higher early mortality risk (3–5).

Non-steroidal anti-inflammatory drugs (NSAIDs), particularly meloxicam, are a promising pharmacological tool in periparturient calf management. Meloxicam preferentially inhibits cyclooxygenase-2 (COX-2), attenuating the prostaglandin-mediated inflammatory and pain response associated with parturition, while preserving the cytoprotective COX-1 pathway (6). Subcutaneous administration of meloxicam (0.5 mg/kg) immediately after birth has demonstrated benefits in improving standing ability in low-vitality calves (7), increasing vigor scores and colostrum intake (8), and reducing pain indicators (9). However, evidence specifically addressing the temporal dynamics of vitality recovery and acid-base normalization, and the moderating role of calving difficulty on these responses, remains limited. Nevertheless, evidence regarding the magnitude and consistency of these benefits remains mixed; some controlled trials have reported only modest or non-significant effects of periparturient meloxicam on short-term clinical outcomes (10), underscoring the need for a prospective evaluation of the conditions under which a clinically meaningful benefit can be expected.

The acid-base status of neonatal calves reflects the cumulative effect of perinatal hypoxia, respiratory adaptation, and metabolic recovery in the first hours of life. Key parameters measured by the ABL800 Basic blood gas analyzer—including blood pH, partial pressure of carbon dioxide (pCO_2_), partial pressure of oxygen (pO_2_), base excess (BE), bicarbonate (${\text{HCO}}_{3}^{-}$), total carbon dioxide (TCO_2_), hemoglobin (Hb), and the anion gap—collectively characterize the respiratory and metabolic components of perinatal acidosis (4, 5). These parameters are clinically significant because severe respiratory-metabolic acidosis (pH < 7.20, BE < −8 mmol/L) is associated with failure of passive immunity transfer and increased neonatal mortality (3, 11, 12). Dam-level factors, including parity and body condition score (BCS), have been associated with colostrum quality, fetal programming, and offspring vitality (13, 14), but their relationship with early cardiac autonomic variables has not been examined. In particular, although parity and body condition are established correlates of colostrogenesis and offspring vigor, their potential association with indices of early cardiac autonomic function—an upstream physiological correlate of perinatal adaptation—has not previously been investigated in dairy calves. Critically, no previous controlled study has prospectively examined the interaction between periparturient meloxicam administration and calving difficulty on the temporal dynamics of vitality recovery and acid-base normalization over the first 48 h of life.

Heart rate variability (HRV) provides a non-invasive measure of cardiac autonomic nervous system function in dairy cattle (15). Measurements at 5 and 30 min postpartum were selected to represent two distinct phases of early neonatal autonomic adaptation; the acute post-birth period (5 min), during which sympathoadrenal activation is near maximal, and the early stabilization phase (30 min), during which partial autonomic recovery has occurred. The first 2 h after birth represent a critical window for colostrum administration decisions; if early HRV could reliably identify low-vitality calves, it would provide a simple, non-invasive tool for targeting supportive interventions before clinical deterioration becomes apparent.

The main objectives of this study were to evaluate the effect of meloxicam on neonatal vitality dynamics and blood gas and acid-base status over 48 h, including the moderating role of calving difficulty; to characterize the associations of dam-related parameters (parity, BCS, licking duration of the calf, calving difficulty score) with early HRV and blood gas and acid-base parameters; to examine the effect of birth time of day and season on physiological parameters; and to evaluate the predictive value of early cardiac autonomic variables, calving difficulty, and birth weight for low vitality at 2 h postpartum.

## 2 Materials and methods

### 2.1 Ethics statement

All procedures were performed in accordance with the guidelines of the Pest County Government Office, Department of Animal Health (Permit Number: PE/EA/1973-6/2016).

### 2.2 Animals, housing, and experimental design

The study was conducted on a large commercial dairy farm in Hungary with >1,000 lactating Holstein-Friesian cows between 2016 March and 2017 February. Two hundred Holstein-Friesian calves were enrolled. Twin calves (*n* = 14) and calves with posterior presentation (*n* = 6) were excluded *a priori*, yielding 180 enrolled calves. A further 29 were excluded for insufficient cardiac recording quality, leaving *n* = 151 (NSAID *n* = 54, Placebo *n* = 97). From 28 d before calving, preparturient cows and heifers were housed in a pre-calving group pen (45 m × 25 m, 50–60 animals, deep straw bedding). Calves were removed from dams within 2 h of birth and fed colostrum via nipple bottle four times daily (1.65 L per feeding) during the first 48 h. Newborns were housed individually in plastic calf hutches (1.65 m × 1.20 m, CalfOtel Comfort, VDK Products, Moergestel, Netherlands) on straw bedding. This manuscript was prepared in accordance with the ARRIVE 2.0 guidelines for reporting animal research (16).

Immediately after delivery, calves were assigned by a quasi-randomization procedure (even/odd birth date) to subcutaneous injection of either meloxicam (0.5 mg/kg; Melovem, AgriHealth; NSAID group) or visually identical placebo (Control group). Allocation was carried out by a single investigator who prepared the syringes; the injections were visually indistinguishable in volume, color and syringe labeling. All personnel involved in neonatal vitality scoring, blood sampling, HRV recording and data processing were unaware of group allocation, and the allocation key was held separately and released only after database closure. Because assignment followed a fixed calendar rule, the allocation sequence was predictable, and allocation concealment was therefore not ensured. This quasi-randomization approach resulted in an unequal group allocation (NSAID *n* = 54, Placebo *n* = 97) because the calving rate across even and odd birth dates was not perfectly balanced over the study period; this imbalance is acknowledged as a study limitation. The difficulty of calving was recorded using the four-grade scale of Mee et al. (17) as follows: Grade 1 (eutocia) = unassisted calving or calving requiring only slight manual traction by one person without mechanical aid; Grade 2 (light dystocia) = calving requiring moderate traction by one person, with the calf delivered within a short period and without instrumentation; Grade 3 (mild dystocia) = calving requiring considerable traction by two or more persons or the use of a calving jack, with delivery achieved without veterinary intervention; Grade 4 (severe dystocia) = calving requiring veterinary assistance, including prolonged forced extraction, correction of fetal malposture, fetotomy or cesarean section. For mixed model analysis, calving was dichotomized as normal (Grade 1) or dystocic (Grades 2–4). The resulting four analytical subgroups were: NSAID-Normal (*n* = 34), NSAID-Dystocic (*n* = 20), Placebo-Normal (*n* = 69), and Placebo-Dystocic (*n* = 28). The continuous 1–4 scale was used in logistic regression and ROC analyses.

### 2.3 Neonatal vitality scoring

Vitality was scored by a trained observer blinded to treatment using the validated 12-point system based on the suggestion of Szenci (18) at 0, 1, 2, 24, and 48 h postpartum. Six parameters were each scored 0–2: muscle tone, head erection, muscle reflexes, mucous membrane color, heart rate, and sucking drive (4). Calves scoring < 7 were classified as low vitality; 7–12 as normal vitality.

### 2.4 Blood gas and acid-base analysis

Venous blood samples were collected by jugular venipuncture into 2 ml lithium heparin syringes (Blood Gas Monovette LH, Sarstedt, Numbrecht-Rommelsdorf, Germany) with the calf in lateral recumbency, at least 1 min following posture change, simultaneously with vitality scoring at 0, 1, 2, and 24 h postpartum. Sampling was performed by the same two trained operators throughout the experiment. The jugular furrow was clipped only when necessary and disinfected with 70% ethanol; a 20-gauge, 40-mm needle was used and the first drops of blood were discarded to avoid tissue-fluid contamination. Syringes were filled to the nominal 2 ml mark to maintain a constant heparin-to-blood ratio, air bubbles were expelled immediately, the syringe was sealed with a cap and gently rolled for at least 10 s to ensure anticoagulant mixing. Samples were kept at ambient barn temperature in an upright position and transported to the on-farm laboratory within 3 min. All samples were analyzed within 5 min of collection using the ABL800 Basic system (Radiometer Medical ApS, Bronshoj, Denmark). The analyzer was operated under an automated internal quality-control programme with three-level control solutions run at the intervals specified by the manufacturer, and automatic two-point calibration before each measurement cycle; measurements were accepted only when all control values were within the manufacturer-specified ranges. The following parameters were measured directly: blood pH; partial pressure of CO_2_ (pCO_2_; mmHg); partial pressure of O_2_ (pO_2_; mmHg); hemoglobin (Hb; g/L); ionized calcium (Ca^2+^; mmol/L); sodium (Na^+^; mmol/L); potassium (K^+^; mmol/L); and chloride (Cl^−^; mmol/L). The following parameters were calculated by the analyzer: bicarbonate (${\text{HCO}}_{3}^{-}$; mmol/L); total carbon dioxide (TCO_2_; mmol/L); base excess of extracellular fluid (BE; mmol/L); and anion gap (mmol/L), calculated as [Na^+^] – [Cl^−^] – [${\text{HCO}}_{3}^{-}$]. Values of pH, pCO_2_, pO_2_, BE, and TCO_2_ were corrected for the rectal temperature of the calf, measured at each sampling. Metabolic acidosis was defined as pH < 7.20 with BE < −8 mmol/L. Due to insufficient sample volume, lactate was measured in only 45 of 151 calves and was not included in group-level statistical analyses.

### 2.5 Recording and analysis of HRV

Cardiac inter-beat interval (IBI) data were recorded continuously from birth to 48 h post-partum using validated Polar heart rate sensors (Polar Equine T56H transmitter with RS800 CX HR monitor, Polar, Kempele, Finland) with an elastic electrode belt. Electrode sites were covered with ultrasound transmission gel (Aquaultra Blue, MedGel Medical, Barcelona, Spain). Devices were fitted to each calf immediately after vitality scoring, and recording commenced within 2 min of birth.

Data were analyzed using Kubios HRV software (version 2.2; Biomedical Signal Analysis Group, Department of Applied Physics, University of Kuopio, Finland) (19). For artifact correction, the custom filter was set at 0.30, identifying IBIs deviating from the preceding interval by more than 30% as artifacts. For detrending, the smoothness prior method was applied with lambda = 1,000 and *fc* = 0.029 Hz, following established recommendations for cattle HRV analysis (20).

Heart rate variability parameters were calculated in equal 5-min windows for 5 min (the first 5 min of the IBI recording) and the 30-min timepoint (a 5-min window spanning 25–30 min postpartum; hereafter referred to as “30-min HRV”) after delivery. In the time domain, mean heart rate (HR) and the root mean square of successive differences in the consecutive IBIs (RMSSD) were quantified. In the frequency domain, IBI data were subjected to Fast Fourier Transformation for power spectrum analysis (21). Frequency band limits were set as LF: 0.05–0.20 Hz and HF: 0.20–0.58 Hz (15, 20). The LF and HF powers were expressed in normalized units (n.u.), and the LF/HF ratio was calculated as an index of sympathovagal balance.

### 2.6 Additional parameters

Dam parity was recorded as a binary variable (0 = primiparous heifer; 1 = multiparous cow). Body condition score of the dam was assessed using the 5-point scale (22) immediately after calving. Maternal licking behavior was recorded on video within the first hour postpartum. Birth time was categorized into four 6-h slots and birth date into meteorological seasons (Spring: March–May; Summer: June–August; Autumn: September–November; Winter: December–February).

### 2.7 Statistical analysis

All analyses were performed in R 4.3.3 (23). Significance was *P* < 0.05 throughout. The effect of NSAID on vitality score dynamics was evaluated using a linear mixed model (LMM) with repeated measurements per calf. Fixed effects included NSAID treatment, time (5-level factor: 0, 1, 2, 24, 48 h), calving difficulty (binary), and their three-way interaction, with birth weight and sex as covariates; a random intercept per calf was specified. Model selection used likelihood ratio tests (LRT) comparing ML-fitted nested models. Goodness of fit was assessed by marginal (*R*^2^*m*) and conditional (*R*^2^*c*) R-squared values (24). *Post-hoc* contrasts between NSAID and Placebo at each timepoint were FDR-adjusted (25). For the significant three-way interaction in the RMSSD LMM, effect size was additionally quantified using Cohen's *f*, computed from the partial η^2^ of the interaction term as *f* = √[η^2^/(1 – η^2^)]. A *post-hoc* sensitivity power analysis was performed using G^*^Power 3.1 (26), with the observed Cohen's *f*, α = 0.05, and the actual per-cell sample sizes.

Group differences in blood gas and acid-base parameters at each timepoint were assessed using Wilcoxon rank-sum tests. In addition, and to account for the repeated-measures structure of the blood gas dataset, each parameter was analyzed with a linear mixed model analogous to that used for vitality, with treatment, time (4-level factor: 0, 1, 2, 24 h) and their interaction as fixed effects, calving difficulty (binary), calf sex and mean-centered birth weight as covariates, and a random intercept per calf. Fixed effects were tested by likelihood ratio tests comparing ML-fitted nested models. This approach accounts for within-calf correlation over time and reduces reliance on multiple pairwise comparisons. Associations between dam-related parameters and HRV variables, blood gas parameters, and vitality scores were assessed using Spearman rank correlation (two-sided), restricted to the Placebo group only (*n* = 97). The effect of birth time slot and season was assessed using the Kruskal–Wallis test. Correlation analyses were considered exploratory; no correction for multiple comparisons was applied to the Spearman rank correlation matrices, and results from these analyses should be interpreted accordingly.

The primary outcome for the predictor analysis was low neonatal vitality at 2 h (vitality 2 h < 7; *n* = 35 cases, *n* = 116 non-cases). Separate logistic regression models were fitted for each predictor (HR, RMSSD, HF, LF/HF, birth weight, calving difficulty score), adjusted for NSAID group, sex, and mean-centered birth weight. Odds ratios (OR) per SD unit are reported with 95% confidence intervals. ROC curves were constructed using the pROC package (27) with AUC and 95% bootstrap CIs estimated; optimal cut-off values were identified by the Youden index. To determine whether early HRV provides predictive information beyond calving difficulty, a multivariable logistic regression model containing both calving difficulty score and RMSSD at 5 min (adjusted for treatment, sex and mean-centered birth weight) was compared with the corresponding single-predictor models. Nested models were compared by likelihood ratio test, and discrimination was compared as the difference in AUC, with a 95% confidence interval and *P*-value obtained from 2,000 bootstrap resamples.

## 3 Results

### 3.1 Animals and baseline characteristics

Treatment groups did not differ significantly in birth weight (NSAID: 39.1 ± 4.8 kg; Placebo: 38.3 ± 4.7 kg; *P* = 0.49), calving difficulty score (*P* = 0.29), or sex ratio (*P* = 0.76; Table 1). The proportion of calves with low vitality at birth (score < 7) was 35.2% (NSAID) and 35.1% (Placebo; *P* = 0.99).

**Table 1:** Baseline characteristics of the enrolled calvings (mean ± *SD* or *n*, %).

Variable	NSAID (*n* = 54)	Placebo (*n* = 97)	*P*-value
Birth weight (kg)	39.1 ± 4.8	38.3 ± 4.7	0.49
Calving difficulty score (1-4)	2.2 ± 0.8	2.1 ± 0.7	0.29
Difficult calving, *n* (%)	21 (38.9)	30 (31.9)	0.07
Bull, *n* (%)	19 (35.2)	36 (37.1)	0.76
Vitality at birth (0–12)	9.3 ± 2.3	9.8 ± 2.2	0.21
Low vitality at birth (< 7), *n* (%)	19 (35.2)	34 (35.1)	0.99
Dam parity: multiparous, *n* (%)	40 (74.1)	71 (73.2)	0.91
Dam body condition score (1-5)	2.52 ± 0.24	2.51 ± 0.22	0.84

*P*-values from *t*-test, Wilcoxon rank-sum test, or χ^2^ test as appropriate.

### 3.2 Neonatal vitality score dynamics

Vitality scores increased significantly over time in both groups (LRT for time: χ^2^ = 186.4, *P* < 0.001). The NSAID main effect was not significant (χ^2^ = 1.40, *P* = 0.236). A significant three-way interaction among NSAID, time, and calving difficulty was detected (χ^2^ = 85.2, *P* < 0.001; *R*^2^*m* = 0.318, *R*^2^*c* = 0.682; Table 2). *Post-hoc* contrasts showed that NSAID-treated calves had higher vitality scores at 24 h (difference = 0.544, *SE* = 0.243, *t* = 2.24, *P* = 0.026) and 48 h (difference = 0.582, *SE* = 0.243, *t* = 2.39, *P* = 0.017). No group differences were observed at 1 h (*P* = 0.856) or 2 h (*P* = 0.240).

**Table 2:** Results for the linear mixed model for vitality score; fixed effects.

Effect	χ^2^	Df	*P*-value	*R*^2^*m*/*R*^2^*c*
Time (0, 1, 2, 24, 48 h)	186.4	4	< 0.001	–
NSAID treatment	1.40	1	0.236	–
Calving difficulty	0.18	1	0.671	–
Birth weight	2.84	1	0.092	–
Sex	0.94	1	0.332	–
NSAID × time × calving difficulty	85.2	16	< 0.001	0.318/0.682

*R*^2^*m* = marginal; *R*^2^*c* = conditional *R*-squared (24). FDR-adjusted *post-hoc*: NSAID > placebo at 24 h (*P* = 0.026) and 48 h (*P* = 0.017).

### 3.3 Blood gas and acid-base status

**Figure 1:**
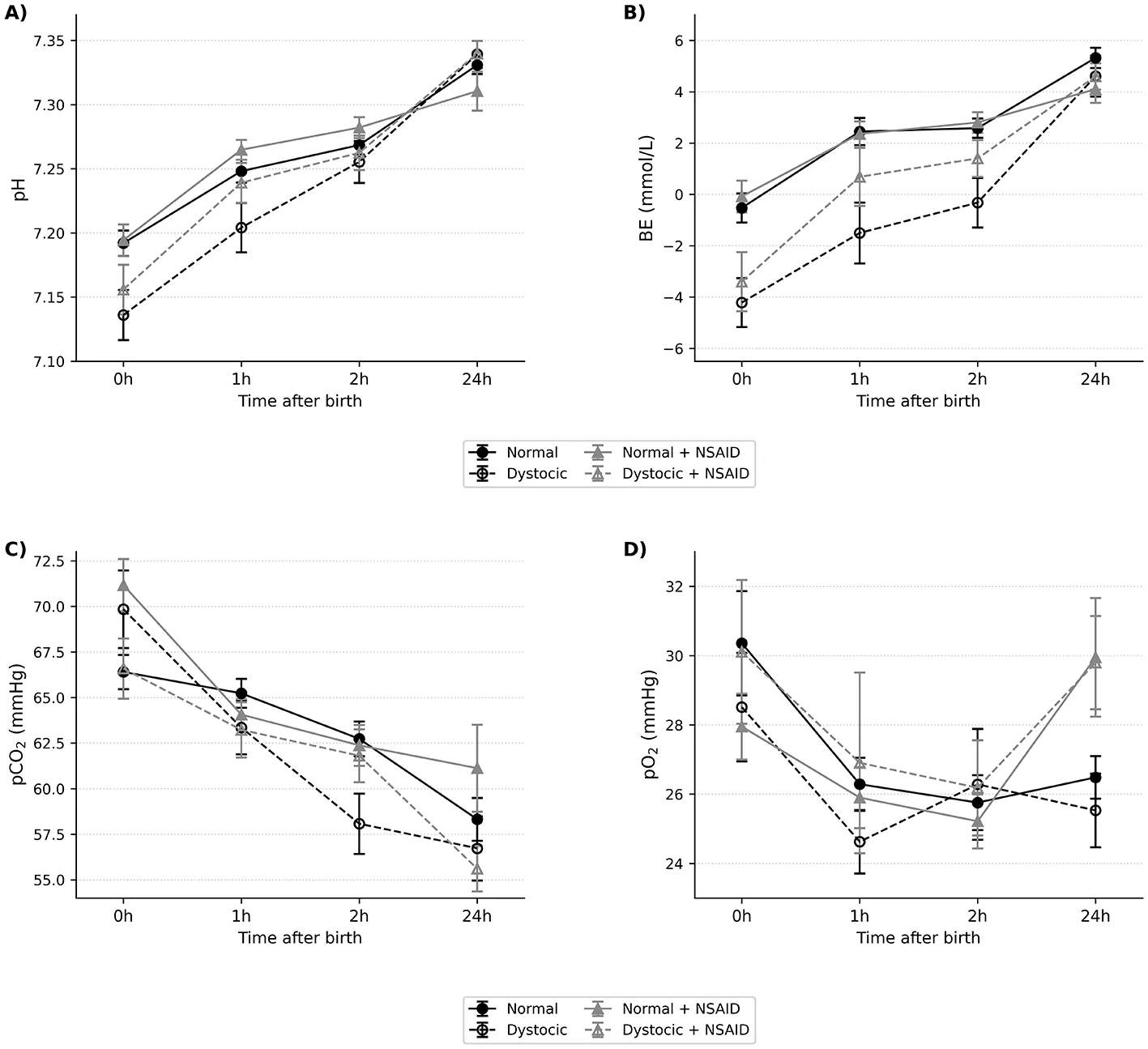
Blood pH **(A)**, base excess **(B)**, pCO_2_ **(C)** and pO_2_ **(D)** in neonatal Holstein-Friesian calves over the first 24 h of life, by treatment group and calving difficulty. Calves received a single subcutaneous dose of meloxicam (0.5 mg/kg; NSAID) or visually identical placebo immediately after delivery; calving was classified as normal (Grade 1) or dystocic (Grades 2–4) using the four-grade scale of Mee et al. (17). Venous blood was sampled at 0, 1, 2 and 24 h postpartum and analyzed within 5 min. Symbols show group means and error bars the standard error of the mean; *n* = 151 calves (NSAID *n* = 54, placebo *n* = 97).

**Figure 2:**
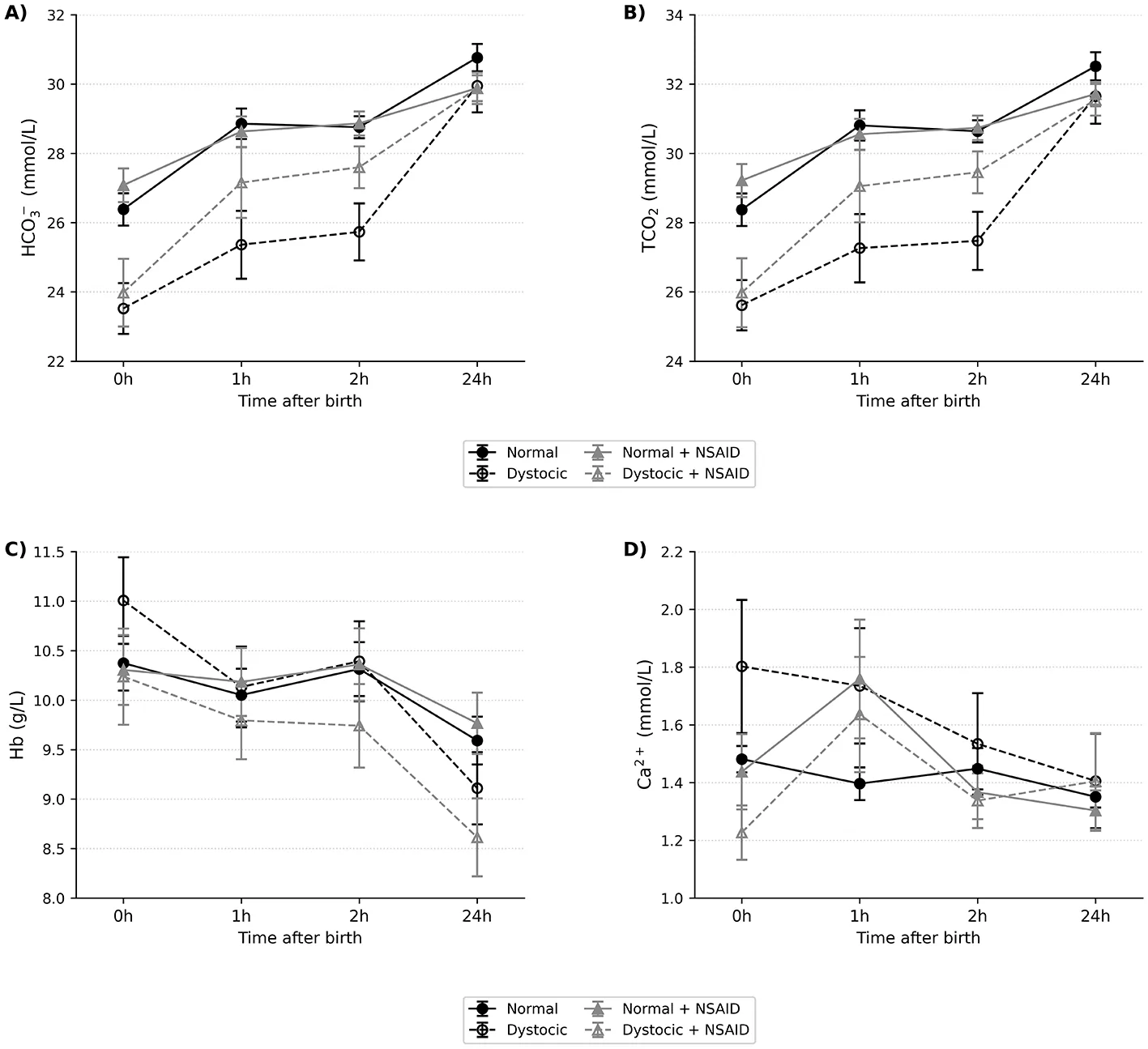
Bicarbonate (${\text{HCO}}_{3}^{-}$, **A**), total carbon dioxide (TCO_2_, **B**), hemoglobin (Hb, **C**) and ionized calcium (Ca^2+^, **D**) in neonatal Holstein-Friesian calves over the first 24 h of life, by treatment group (meloxicam 0.5 mg/kg subcutaneously vs. placebo at birth) and calving difficulty (normal = Grade 1; dystocic = Grades 2–4). Symbols show group means and error bars the standard error of the mean; *n* = 151 calves.

**Figure 3:**
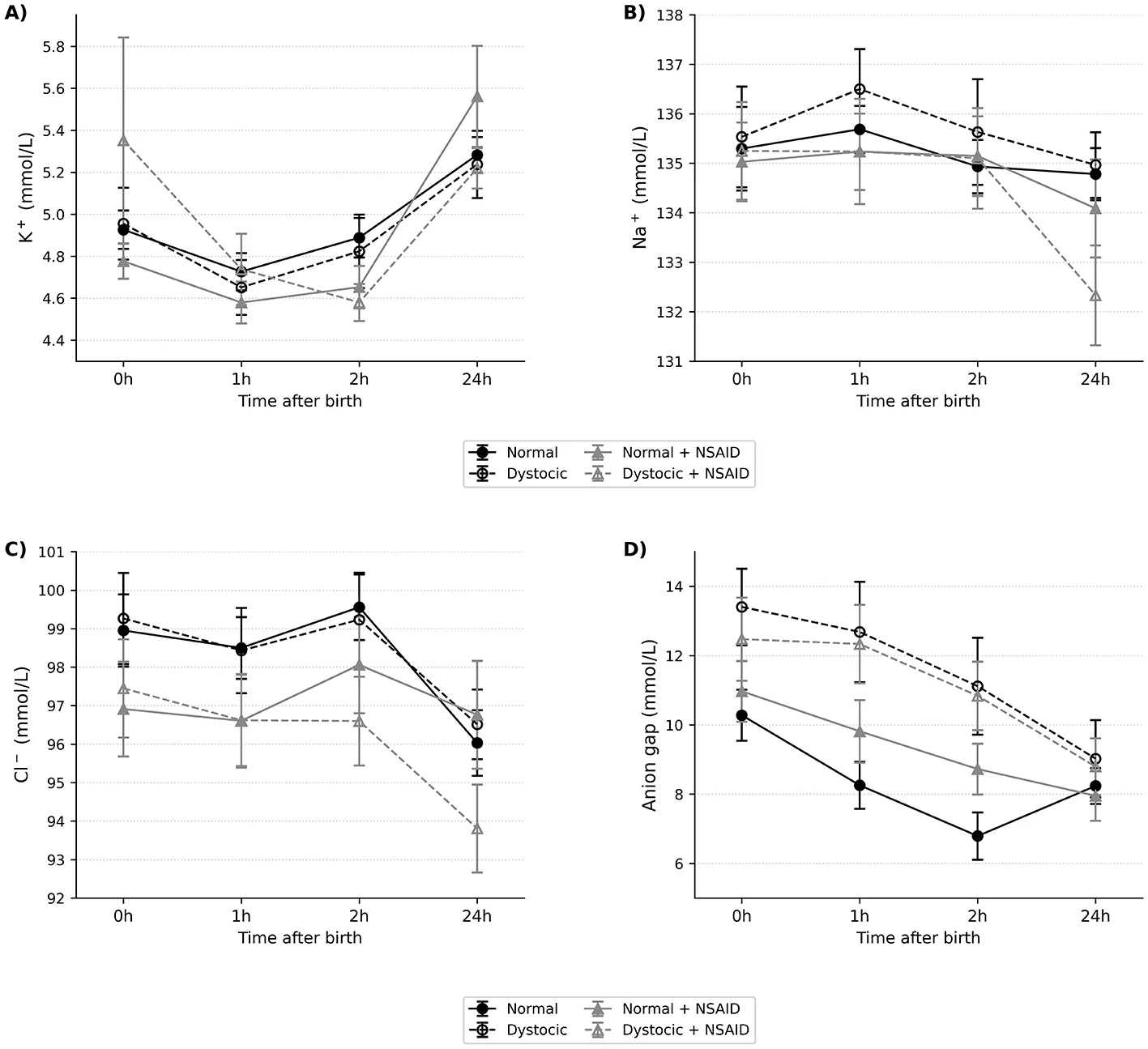
Serum sodium (**A**), potassium (**B**), chloride (**C**) and anion gap (**D**) in neonatal Holstein-Friesian calves over the first 24 h of life, by treatment group (meloxicam 0.5 mg/kg subcutaneously vs. placebo at birth) and calving difficulty (normal = Grade 1; dystocic = Grades 2–4). Anion gap was calculated as [Na^+^] – [Cl^−^] – [${\text{HCO}}_{3}^{-}$]. Symbols show group means and error bars the standard error of the mean; *n* = 151 calves.

Blood gas and acid-base parameters at 0 h reflected a mild combined respiratory-metabolic acidosis in all groups (Table 3; pH and blood gas dynamics in Figure 1, ${\text{HCO}}_{3}^{-}$, Hb, TCO_2_ and Ca^2+^ in Figure 2, electrolytes in Figure 3).

**Table 3:** Blood gas and acid-base parameters by treatment group at 0 and 24 h (mean ± *SD*).

Parameter	NSAID 0 h	Placebo 0 h	NSAID 24 h	Placebo 24 h	P^*^24 h
pH	7.17 ± 0.08	7.18 ± 0.09	7.33 ± 0.05	7.34 ± 0.05	0.469
pCO_2_ (mmHg)	69.9 ± 8.9	67.1 ± 9.2	57.7 ± 7.0	57.9 ± 8.3	0.998
pO_2_ (mmHg)	30.4 ± 14.3	30.4 ± 14.4	42.1 ± 13.8	41.8 ± 13.6	0.871
BE (mmol/L)	−1.89 ± 4.63	−1.71 ± 4.99	4.45 ± 2.53	5.30 ± 3.09	0.049^*^
${\text{HCO}}_{3}^{-}$ (mmol/L)	25.5 ± 3.9	25.4 ± 4.0	32.1 ± 2.8	32.8 ± 3.1	0.098
TCO_2_ (mmol/L)	28.0 ± 3.9	27.5 ± 4.0	31.7 ± 2.0	32.2 ± 3.6	0.112
Hb (g/L)	10.4 ± 2.3	10.4 ± 2.2	10.1 ± 1.9	10.0 ± 2.0	0.743
Anion gap (mmol/L)	11.3 ± 6.2	11.3 ± 6.2	9.8 ± 3.2	9.5 ± 3.1	0.621
K^+^ (mmol/L)	4.82 ± 0.64	4.92 ± 0.77	5.24 ± 0.43	5.19 ± 0.46	0.876
Na^+^ (mmol/L)	135.2 ± 3.9	134.4 ± 4.9	133.8 ± 4.3	134.3 ± 5.8	0.987
Cl^−^ (mmol/L)	98.3 ± 5.4	97.8 ± 7.3	96.0 ± 5.9	95.5 ± 6.4	0.724
Ca^2+^ (mmol/L)	1.28 ± 0.30	1.52 ± 0.41	1.29 ± 0.36	1.36 ± 0.32	0.005^*^

BE, base excess of ECF; ${\text{HCO}}_{3}^{-}$ = bicarbonate; TCO_2_, total CO_2_; Hb, hemoglobin. P^*^ = *P*-value for NSAID vs. placebo at 24 h. ^*^P < 0.05. Group differences at 0 h: Ca^2+^ *P* = 0.006; all other parameters *P* > 0.10.

All parameters improved significantly from 0 to 24 h in both groups (Figures 1–3). Blood pH increased from 7.17–7.18 at 0 h to 7.24 by 1 h, 7.29 by 2 h, reaching 7.33 at 24 h. The only significant treatment difference was at 24 h, where BE was lower in NSAID-treated calves (NSAID: 4.45 ± 2.53 vs. Placebo: 5.30 ± 3.09 mmol/L; *P* = 0.049; Table 3). Ionized calcium (Ca^2+^) was lower in NSAID-treated calves both at 0 h (1.28 ± 0.30 vs. 1.52 ± 0.41 mmol/L; *P* = 0.006) and at 24 h (1.29 ± 0.36 vs. 1.36 ± 0.32 mmol/L; *P* = 0.005). Dystocic calves showed lower pH (*P* = 0.031), lower pO_2_ (*P* = 0.044), lower BE (*P* = 0.028), and higher pCO_2_ (*P* = 0.037) at 0 h; these differences resolved by 2 h.

In the repeated-measures mixed models, time was a highly significant predictor for all blood gas and acid-base parameters (all *P* < 0.001), and calving difficulty remained significantly associated with pH, pCO_2_, BE, ${\text{HCO}}_{3}^{-}$, TCO_2_ and anion gap (all *P* < 0.05). The main effect of treatment was not significant for any parameter except for Cl^−^ (*P* = 0.020), and the treatment-by-time interaction was significant only for pO_2_ (*P* = 0.002) and ionized calcium (*P* = 0.025). The between-group difference in BE observed at 24 h in the pairwise comparison was therefore not supported as a systematic treatment effect in the repeated-measures framework (main effect *P* = 0.446; interaction *P* = 0.119).

### 3.4 Effects of dam-related parameters, birth time and season

**Figure 4:**
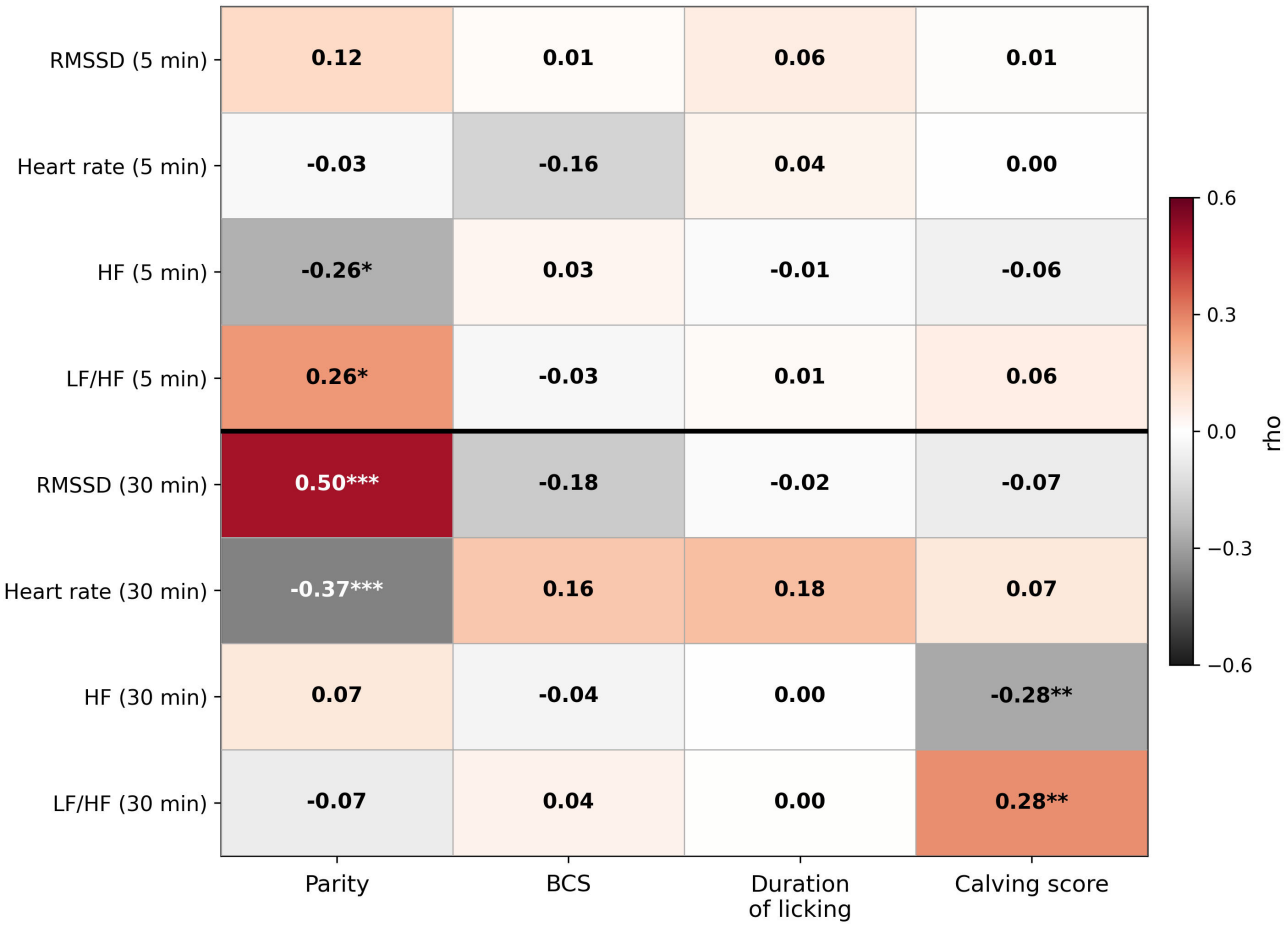
Heatmap of Spearman rank correlation coefficients between dam-related parameters (parity, body condition score, duration of maternal licking during the first hour, and calving difficulty score on the four-grade scale of Mee et al. 17) and heart rate variability variables measured in 5-min windows at 5 and 30 min postpartum. Analyses were restricted to placebo-treated calves (*n* = 97) thus, associations reflect unmedicated physiology. Cell values show the correlation coefficient rho, with warm colors indicating positive and cool colors negative associations; *P < 0.05, **P < 0.01, ***P < 0.001. Correlations were exploratory and not corrected for multiple testing.

**Figure 5:**
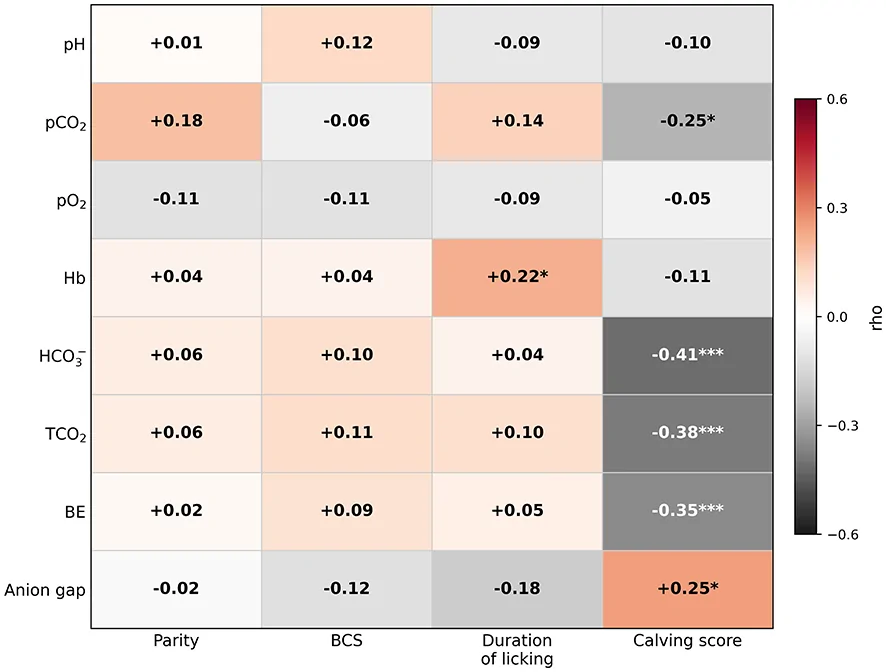
Heatmap of Spearman rank correlation coefficients between dam-related parameters (parity, body condition score, duration of maternal licking during the first hour, and calving difficulty score) and venous blood gas, acid-base and electrolyte parameters measured at 2 h postpartum. Analyses were restricted to placebo-treated calves (*n* = 97). Cell values show the correlation coefficient rho, with warm colors indicating positive and cool colors negative associations; *P < 0.05, ***P < 0.001. Correlations were exploratory and not corrected for multiple testing.

**Figure 6:**
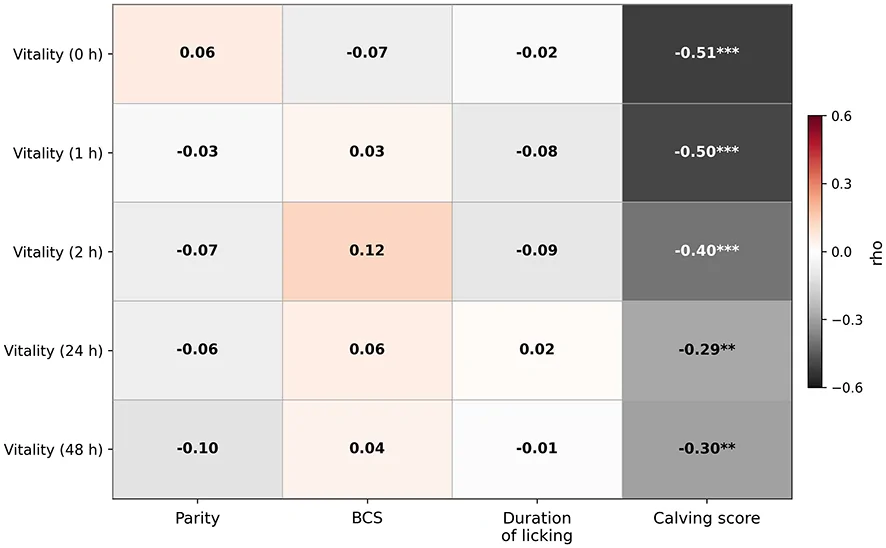
Heatmap of Spearman rank correlation coefficients between dam-related parameters (parity, body condition score, duration of maternal licking during the first hour, and calving difficulty score) and neonatal vitality score, assessed on a validated 12-point scale at 0, 1, 2, 24 and 48 h postpartum. Analyses were restricted to placebo-treated calves (*n* = 97). Cell values show the correlation coefficient rho, with warm colors indicating positive and cool colors negative associations; **P < 0.01, ***P < 0.001. Correlations were exploratory and not corrected for multiple testing.

In the Placebo group, parity was significantly associated with HRV at 30 min (RMSSD: rho = 0.50, *P* < 0.001; HR: rho = −0.37, *P* < 0.001), whereas no significant associations were found at 5 min (all *P* > 0.10). Parity was also positively associated with blood gas parameters at 2 h: pH (rho = 0.31, *P* = 0.002), BE (rho = 0.28, *P* = 0.006), and ${\text{HCO}}_{3}^{-}$ (rho = 0.26, *P* = 0.011). Calving difficulty score was significantly and negatively associated with vitality at all timepoints (rho = −0.47 to −0.54, all *P* < 0.001) and with blood acid-base parameters at 2 h. Body condition score showed no significant associations with HRV variables (all *P* > 0.14) or vitality scores (all *P* > 0.94). Licking duration showed a positive association with vitality at 0 h (rho = 0.22, *P* = 0.030). Heatmaps of these associations are shown in Figures 4–6.

The birth time slot did not significantly affect any blood acid-base or HRV parameters (all *P* = 0.295–0.985). Among seasonal comparisons, only pO_2_ at 0 h differed significantly across seasons (Kruskal–Wallis *H* = 14.020, df = 3, *P* = 0.003), with lower values in Summer (25.1 ± 12.3 mmHg) compared to Autumn (32.1 ± 14.8 mmHg) and Winter (34.2 ± 13.9 mmHg).

### 3.5 Results of predictor analysis

Of the 35 calves with low vitality at 2 h, RMSSD at 5 min was the only HRV variable with a significant association in adjusted logistic regression (OR = 0.484 per SD unit, 95% *CI* 0.25–0.79, *P* = 0.004; Table 4). No other HRV variable at 5 or 30 min was significant (all *P* > 0.20). Calving difficulty score remained the strongest predictor: each unit increase in difficulty grade was associated with 3.5-fold higher odds of low vitality at 2 h (OR = 3.54, 95% *CI* 2.44–5.13, *P* < 0.001).

**Table 4:** Predictors of low vitality at 2 h postpartum (vitality 2 h < 7; *n* = 35 cases, *n* = 116 non-cases).

Predictor	OR (per SD)	95% CI low	95% CI high	*P*-value
RMSSD 5 min	0.484	0.25	0.79	0.004
HR 5 min	1.201	0.80	1.83	0.376
HF 5 min	1.033	0.63	1.60	0.850
LF/HF 5 min	0.987	0.58	1.54	0.926
RMSSD 30 min	1.015	0.47	1.48	0.999
HR 30 min	1.279	0.85	2.11	0.221
HF 30 min	1.007	0.52	1.48	0.971
LF/HF 30 min	0.840	0.55	1.28	0.311
Birth weight (kg)	1.000	0.95	1.05	0.970
Calving difficulty (1-4)	3.54	2.44	5.13	< 0.001

Separate logistic regression models adjusted for NSAID treatment, sex, and mean-centered birth weight. OR, odds ratio; CI, confidence interval.

**Figure 7:**
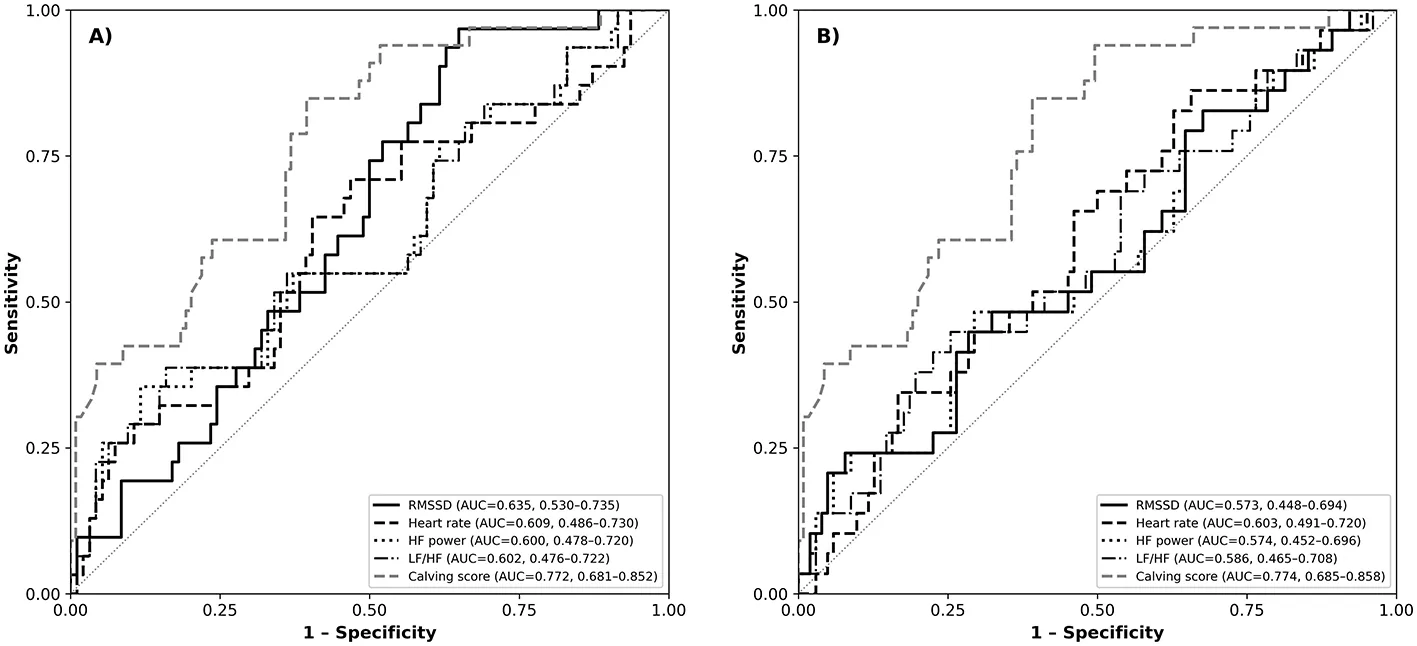
Receiver operating characteristic (ROC) curves for the prediction of low neonatal vitality at 2 h postpartum (vitality score <11 on a 12-point scale; *n* = 35 cases, *n* = 116 non-cases) in Holstein-Friesian calves. **Panel A** shows heart rate variability (HRV) variables derived from a 5-min recording window starting at birth; **Panel B** shows the corresponding variables from a 5-min window spanning 25–30 min postpartum. Birth weight and calving difficulty score (four-grade scale of Mee et al. 17) are shown in both panels for comparison. HRV variables are mean heart rate (HR), root mean square of successive differences (RMSSD), high-frequency power (HF) and low- to high-frequency power ratio (LF/HF). Areas under the curve (AUC) with 95% bootstrap confidence intervals are given in the legend; the dashed diagonal denotes chance-level discrimination (AUC = 0.50). An AUC above 0.70 is conventionally regarded as clinically useful discrimination.

The ROC analysis showed that RMSSD at 5 min had the highest discriminative value among HRV variables (AUC = 0.635, 95% CI 0.530–0.735) (Table 5; ROC curves in Figure 7). Calving difficulty score remained the strongest single predictor (AUC = 0.772, 95% CI 0.681–0.852).

**Table 5:** Results of the ROC analysis; prediction of low vitality at 2 h (vitality 2 h < 7; *n* = 35 cases, *n* = 116 non-cases).

Predictor	AUC	CI low	CI high	Sensitivity	Specificity
RMSSD 5 min	0.635	0.530	0.735	0.97	0.35
HR 5 min	0.609	0.486	0.730	0.71	0.53
HF 5 min	0.600	0.478	0.720	0.35	0.88
LF/HF 5 min	0.602	0.476	0.722	0.39	0.84
RMSSD 30 min	0.573	0.448	0.694	0.45	0.72
HR 30 min	0.603	0.491	0.720	0.86	0.34
HF 30 min	0.574	0.452	0.696	0.48	0.71
LF/HF 30 min	0.586	0.465	0.708	0.45	0.75
Birth weight (kg)	0.424	0.304	0.544	0.17	0.89
Calving difficulty (1–4)	0.772	0.681	0.852	0.85	0.61

AUC, area under the ROC curve; CI, 95% bootstrap confidence interval. AUC > 0.70 is generally considered clinically useful discrimination (35). All models adjusted for NSAID treatment, sex, and mean-centered birth weight.

In the multivariable model combining calving difficulty score and RMSSD at 5 min, calving difficulty remained significantly associated with low vitality at 2 h, whereas RMSSD was no longer significant. Adding RMSSD to the calving difficulty model improved model fit (likelihood ratio test, χ^2^ = 4.39, *P* = 0.036) and lowered the Akaike information criterion, but produced no meaningful gain in discrimination: the AUC increased from 0.724 (95% CI 0.618–0.818) for calving difficulty alone to 0.751 (95% CI 0.652–0.843) for the combined model, a difference of +0.027 (95% CI −0.025 to 0.075; *P* = 0.300). Conversely, adding calving difficulty to the RMSSD model produced a substantial improvement (χ^2^ = 11.97, *P* < 0.001).

## 4 Discussion

The principal findings were: (1) meloxicam was associated with higher vitality at 24 and 48 h in a calving difficulty-dependent manner, without affecting blood acid-base parameters at birth; (2) BE was lower in NSAID-treated calves at 24 h; (3) in unmedicated calves, parity and calving difficulty were associated with HRV at 30 min and acid-base status at 2 h; (4) birth time of day did not affect physiological parameters, while season modestly influenced pO_2_; and (5) RMSSD at 5 min was significantly but weakly associated with low vitality at 2 h (AUC = 0.635), whereas calving difficulty was the dominant predictor (AUC = 0.772).

All calves exhibited the characteristic combined respiratory-metabolic acidosis immediately after birth, with mean pH of 7.17, pCO_2_ of 68 mmHg, and BE of approximately −1.8 mmol/L, consistent with physiological ischemia in the fetoplacental unit during delivery (1). These values remained above the critical threshold of pH < 7.20 and BE < −8 mmol/L (28) for life-threatening neonatal asphyxia. The mean anion gap of 11.3 mmol/L lies within the reference interval and is therefore compatible with a normal anion gap pattern. We emphasize, however, that in the absence of systematic lactate measurement the relative contributions of hyperchloraemia and lactic acidosis cannot be quantified in the present dataset. A definitive attribution would require either systematic lactate determination or the application of a simplified strong ion approach (for example, calculation of the apparent strong ion difference), neither of which was possible here. The characterization of the metabolic component is accordingly restricted to the measured anion gap and electrolyte values, and no inference is drawn as to whether the acidosis was predominantly hyperchloraemic or lactate-related.

Dystocic calves showed significantly lower pH and pO_2_ and higher pCO_2_ at 0 h, consistent with previous reports of dystocia-related respiratory acidosis (2, 29, 30) and with the findings of Homerosky et al. (12).

The absence of a birth-time-of-day effect suggests that circadian variation in attendance and intervention timing was not a meaningful source of physiological heterogeneity in this management system. The modest seasonal effect, confined to pO_2_ and most pronounced in summer, is more plausibly attributable to ambient heat load and its impact on placental perfusion and fetal oxygenation (31, 32) than to seasonal differences in obstetric management, and supports including season as a covariate in future studies.

The significant three-way interaction for vitality, peaking at 24–48 h, is consistent with the pharmacokinetic profile of subcutaneous meloxicam in cattle (6): plasma levels sufficient for anti-inflammatory effect are reached 1–2 h after injection, with a half-life of approximately 26 h (33). The delayed divergence between groups mirrors findings by Kovács et al. (7) and Murray et al. (8). The effect being most pronounced in dystocic calves is biologically coherent: greater COX-2-mediated prostaglandin production in response to obstetrical trauma provides greater substrate for NSAID-mediated attenuation.

The lower BE in NSAID-treated calves at 24 h (4.45 vs. 5.30 mmol/L) requires careful interpretation. The absolute difference of 0.85 mmol/L is small relative to the measurement range and to the within-group standard deviation, both group means lie well within the physiological reference interval, and the difference was not supported by the repeated-measures model. It should therefore be regarded as statistically detectable but of negligible biological or clinical relevance, and it does not indicate impaired acid-base recovery in treated calves. One possible contributory mechanism is that greater physical activity and metabolic demand in more vigorous NSAID-treated calves generate lactate and consume bicarbonate at a faster rate, but this remains speculative in the absence of lactate data.

The lower ionized calcium concentration in NSAID-treated calves at both 0 h (1.28 vs. 1.52 mmol/L) and 24 h (1.29 vs. 1.36 mmol/L) is an unexpected finding. Both values fall within or close to the lower physiological reference range for neonatal calves (1.20–1.40 mmol/L), and the 24-h difference of 0.07 mmol/L is unlikely to be of clinical consequence. Importantly, the difference was already present at 0 h, that is, before meloxicam could plausibly have exerted a pharmacological effect, which indicates that it most likely reflects a baseline imbalance not equilibrated by the quasi-randomization procedure rather than a treatment effect. We therefore refrain from proposing a mechanistic explanation, and note only that replication in a fully randomized trial with pre-calving maternal Ca^2+^ monitoring would be required before any causal interpretation is entertained.

In unmedicated calves, dam parity was positively associated with offspring vagal tone at 30 min, as reflected by RMSSD, and with acid-base status at 2 h. To our knowledge, this is the first report of a direct relationship between dam parity and neonatal cardiac autonomic tone in dairy calves. The stronger association at 30 min compared to 5 min suggests that the parity effect emerges during the early recovery phase. Because parity and calving ease are themselves related, obstetric factors may be the pathway through which parity influences neonatal cardiac autonomic tone, although the present design cannot separate the two. The modest but positive correlation between licking duration and vitality at 0 h is consistent with Kovács et al. (4) and with the general literature supporting the beneficial thermoregulatory and respiratory effects of early maternal contact (34). The significant negative correlation between calving difficulty score and vitality at all timepoints confirms that obstetrical difficulty is the dominant dam-level predictor of neonatal functional status.

The RMSSD at 5 min was the only significant HRV predictor, while no 30 min variable achieved significance. Lower RMSSD at 5 min in calves with subsequent low vitality may reflect more severe perinatal compromise, but the AUC of 0.635 indicates that the association is not clinically useful in isolation. This is consistent with Homerosky et al. (12) and Clapp et al. (34), who showed that HRV reflects cumulative welfare compromise in dairy calves, while extreme autonomic instability in the immediate neonatal period limits single-timepoint measurements as predictors. The Youden-optimal cut-off illustrates the problem: sensitivity was 0.97 but specificity only 0.35, so the threshold would trigger unnecessary intervention in roughly two-thirds of unaffected calves.

The OR of 3.54 per unit increase in calving difficulty score confirms calving ease as the principal determinant of compromised neonatal adaptation (3, 10, 29), and indicates that targeted intervention at birth is most justified for calves born after obstetrical assistance. The comparison is decisive from a triage perspective: calving difficulty is recorded routinely at every parturition, requires no equipment and is available immediately, whereas RMSSD requires continuous ECG-derived recording for lower discriminative ability. A multivariable analysis confirmed this directly: adding RMSSD at 5 min to a model containing calving difficulty improved statistical fit but not discrimination (ΔAUC +0.027, 95% CI −0.025 to 0.075). This dissociation between improved fit and unchanged discrimination is characteristic of a predictor carrying genuine but weak information. Early HRV therefore cannot be recommended as a stand-alone or supplementary triage tool in this setting, and calving difficulty-based risk stratification should be prioritized in on-farm decision-making.

Several limitations of the present study warrant consideration. First, and most importantly, group allocation followed a quasi-randomization rule (even/odd birth date) rather than true randomization. Such a rule is deterministic and, once known, predictable, so allocation concealment cannot be guaranteed even when outcome assessors are blinded; if any person involved in enrolment had been aware of the rule, differential enrolment could in principle have occurred. The procedure also produced unequal group sizes (NSAID *n* = 54, Placebo *n* = 97), which reduces statistical power for between-group contrasts and indicates that calvings were not evenly distributed across even and odd dates. Two observations suggest that residual imbalance was not fully eliminated: ionized calcium differed between groups already at 0 h, and group sizes differed substantially. Although the groups did not differ in birth weight, calving difficulty or sex ratio, the possibility of unmeasured confounding cannot be excluded, and all between-group comparisons should be read as associations within a quasi-experimental design rather than as unbiased causal estimates. Second, data were collected on a single commercial Holstein-Friesian farm, which constrains generalizability to other breeds, housing systems, and management conditions. The herd was a large, intensively managed unit with continuous calving surveillance, trained calving assistance and a standardized colostrum protocol; in herds with less intensive supervision, later obstetrical intervention or different colostrum management, both the baseline vitality distribution and the magnitude of any meloxicam effect could differ. Similarly, the observed distribution of calving difficulty grades reflects the obstetric practice of this particular farm. The findings should therefore be regarded as hypothesis-generating for other production systems, and multi-herd replication is needed before extrapolation. Third, venous lactate was measured in only 45 of 151 calves due to insufficient sample volume, precluding group-level lactate analysis and preventing the application of a strong ion (Stewart) approach to characterize the metabolic component of the acidosis. Fourth, HRV data were collected under field conditions; although the custom artifact filter (30% threshold) was applied, the proportion of recording epochs with excessive artifact rate was not reported, and autonomic instability in the immediate neonatal period may have reduced the reliability of single-timepoint HRV metrics. Fifth, the present study did not include follow-up beyond 48 h, and potential longer-term effects of periparturient meloxicam on passive immune transfer efficiency and early morbidity could not be assessed.

The principal strengths of the study are its prospective, placebo-controlled design with blinded outcome assessment; the relatively large number of calves for a neonatal physiological study; the combination of clinical scoring, blood gas and acid-base analysis and continuous cardiac autonomic monitoring in the same animals; the dense sampling schedule over the first 48 h, which allowed the temporal dynamics of recovery to be characterized rather than a single endpoint; and the inclusion of dam-level covariates that are rarely recorded alongside neonatal outcomes. The explicit evaluation of whether early HRV adds predictive value beyond a routinely recorded clinical variable is, to our knowledge, novel in this species and provides a directly actionable answer for on-farm practice.

## 5 Conclusions

A single subcutaneous dose of meloxicam administered at birth was associated with higher neonatal vitality at 24 and 48 h in Holstein-Friesian calves, with the effect most pronounced in calves born after obstetrical assistance. Blood gas and acid-base parameters at birth were not affected by treatment, suggesting that meloxicam does not directly reverse perinatal acidosis but may modulate the functional recovery trajectory. Base excess at 24 h was modestly lower in NSAID-treated calves; this difference was small, was not supported by the repeated-measures analysis, and is unlikely to be of clinical relevance. Dam parity was significantly associated with early cardiac autonomic tone and acid-base status at birth. Birth time of day did not influence physiological parameters, while pO_2_ at birth was modestly but significantly lower in summer-born calves. The RMSSD at 5 min postpartum was the only HRV variable with a statistically significant association with low vitality at 2 h, though without clinically useful predictive accuracy and without incremental value beyond calving difficulty; calving difficulty score was the strongest predictor. Taken together, these findings suggest that targeted periparturient NSAID administration may be a useful welfare intervention in assisted calvings, and indicate that calving difficulty, rather than early cardiac autonomic status, is the primary determinant of compromised neonatal vitality in dairy calves. Given the quasi-randomized allocation and the single-herd setting, these conclusions should be regarded as provisional pending confirmation in a fully randomized, multi-herd trial.

## Data Availability

The raw data supporting the conclusions of this article will be made available by the authors, without undue reservation.
